# Unicortical Versus Bicortical Screw Fixation for Medial Malleolus Fractures: A Retrospective Comparative Study

**DOI:** 10.7759/cureus.97764

**Published:** 2025-11-25

**Authors:** Muhammad Irfan Akram, Vishal Kumar, Yaser Hasan, Sharon Jafrin Surali Sharfudeen

**Affiliations:** 1 Trauma and Orthopaedics, Ipswich Hospital, Ipswich, GBR; 2 Trauma and Orthopaedics, King's College Hospital National Health Service (NHS) Foundation Trust, London, GBR; 3 Orthopaedic Surgery, National University Hospital, Singapore, SGP

**Keywords:** ankle and foot, ankle fracture management, ankle trauma, bimalleolar ankle fracture, foot and ankle reconstruction, medial ankle, open reduction fixation of ankle fracture, posterior ankle, screw fixation, trauma and orthopaedic

## Abstract

Ankle fractures are common lower extremity injuries often involving the medial malleolus, a critical stabilizer of the ankle mortise. Screw fixation is the standard treatment, but the choice between unicortical and bicortical screw techniques remains debated. This retrospective study evaluates clinical outcomes of unicortical versus bicortical screw fixation in medial malleolus fractures over a four-year period at a Level 1 trauma center. In this study, 137 adult patients were included, with 95 undergoing unicortical fixation and 42 bicortical fixation. The primary outcome was early radiological union at three months. Delayed union rates were similar between groups (unicortical 9%, bicortical 7%, p=0.656). No significant differences in complications or secondary procedures were observed. Despite biomechanical advantages of bicortical screws, our data suggests comparable clinical effectiveness for both techniques. Further prospective studies with longer follow-up and functional outcomes are warranted.

## Introduction

Ankle fractures are among the most prevalent lower limb injuries, frequently resulting from low-energy rotational trauma that forces the talus against the surrounding bony structures [1]. These mechanisms can produce a variety of fracture patterns, often involving the medial malleolus, a key stabilizer of the ankle mortise. Proper reduction and fixation of the medial malleolus are crucial for restoring joint congruence and preventing long-term complications such as chronic pain or post-traumatic arthritis [2].

Screw fixation remains a standard method for stabilizing medial malleolar fractures. However, the choice between unicortical and bicortical screw placement continues to generate discussion in orthopedic practice. While bicortical fixation is thought to provide superior mechanical stability through dual cortical engagement [3], concerns about potential risks - such as soft tissue irritation or neurovascular injury - have limited its universal adoption. Conversely, unicortical fixation may be less invasive but could theoretically compromise stability in certain fracture patterns.

This retrospective study evaluates clinical outcomes of unicortical versus bicortical screw fixation in medial malleolus fractures over a four-year period at a Level 1 trauma center. In the study, 137 adult patients were included, with 95 undergoing unicortical fixation and 42 bicortical fixation. Specifically, we examine radiographic union rates over a four-year period to assess whether bicortical fixation offers measurable benefits in routine clinical practice.

## Materials and methods

This retrospective study was conducted at a Level 1 trauma center in London. Data were collected for ankle fracture fixations over a four-year period (2019-2023). Inclusion criteria were adult patients treated with screw fixation only and with radiological follow-up exceeding three months. Only primary fixations were included. Exclusion criteria included medial malleolus fractures fixed with screw and plate constructs, patients younger than 18 years, neuropathic patients, pathological fractures, and radiological follow-up of less than three months.

Collected data included patient demographics, comorbidities, injury patterns, surgical details, and radiological outcomes. The radiological union was assessed by looking at cortical continuity, bridging trabeculae, and absence of fracture line.

The primary outcome of the study is early radiological union at three months.

Categorical variables (e.g., union status, smoking, diabetes, and injury type) were compared using the chi-square test (χ²). Continuous data (non-weight-bearing duration and radiological follow-up) are expressed as mean±standard deviation (SD) with ranges. A significance threshold of p<0.05 was adopted.

## Results

A total of 137 adult patients with a wide range of ankle fractures (Table 1) and a minimum of three months of radiological follow-up met the inclusion criteria. Of these, 95 patients underwent unicortical fixation, while 42 received bicortical fixation.

**Table 1:** Type fractures according to AO classification The data has been presented as AO classification fracture type, N=numbers and %. AO: Arbeitsgemeinschaft für Osteosynthesefragen (Association of the Study of Internal Fixation).

AO Type	Unicortical (n=95)	Bicortical (n=42)	Total
A3.1	10 (10.5%)	1 (2.4%)	11
B3.2	19 (20.0%)	4 (9.5%)	23
B2.3	4 (4.2%)	6 (14.3%)	10
C2.3	7 (7.4%)	7 (16.7%)	14
C2.2	11 (11.6%)	1 (2.4%)	12
B3.1	1 (1.1%)	0	1
B3.3	23 (24.2%)	9 (21.4%)	32
C1.2	3 (3.2%)	5 (11.9%)	8
B2.2	14 (14.7%)	5 (11.9%)	19
A3.2	1 (1.1%)	0	1
A2.1	2 (2.1%)	0	2
C1.3	0	2 (4.8%)	2
A2.3	0	1 (2.4%)	1
A2.2	0	1 (2.4%)	1

Comorbidity analysis demonstrated no significant association between fixation type and smoking status (p=0.353) or diabetes mellitus (p=0.963). Comparison of injury type revealed a higher proportion of open injuries in the bicortical group (38.1%) compared to the unicortical group (22.1%), but this difference did not reach statistical significance (p=0.052) (Table 2).

**Table 2:** Comparison of comorbidities, injury type, non-weight-bearing status (NWB) and follow-up between fixation groups χ² test (with degrees of freedom) used for categorical comparisons; values shown as n (%); p<0.05 considered significant.

	Unicortical (n=95)	Bicortical (n=42)	Total	χ² (df)	p- value
Smoking					
No	90 (94.7%)	38 (90.5%)	128 (93.4%)	0.86 (1)	0.353
Yes	5 (5.3%)	4 (9.5%)	9 (6.6%)		
Diabetes					
No	88 (92.6%)	39 (92.9%)	127 (92.7%)	0 (1)	0.963
Yes	7 (7.4%)	3 (7.1%)	10 (7.3%)		
Injury type					
Closed	74 (77.9%)	26 (61.9%)	100 (73%)	3.78 (1)	0.052
Open	21(22.1%)	16 38.1%)	37 (27%)		
NWB (weeks)	6.16 ± 1.72 (range 2-17)	5.83 ± 0.76 (range 2-6)			NA
Radiological follow-up (months)	9.84 ± 9.25 (range 3-42)	8.37 ± 6.74 (range 3-36)			NA

Both groups followed a standardized postoperative protocol. The mean non-weight-bearing duration was 6.16±1.72 weeks (range 2-17 weeks) in the unicortical group and 5.83±0.76 weeks (range 2-6 weeks) in the bicortical group. The mean radiological follow-up duration was 9.84±9.25 months (range 3-42 months) in the unicortical group and 8.37±6.74 months (range 3-36 months) in the bicortical group (Table 2).

The primary outcome was fracture union at three months. Union was achieved in 90.5% (86/95) of the unicortical group and 92.9% (39/42) of the bicortical group, with no significant difference between groups (p=0.656) (Table 3). The study did not identify significant differences.

**Table 3:** Comparison of radiological union of the fracture at three months between fixation groups χ² test (with degrees of freedom) used for categorical comparisons; values shown as n (%); p<0.05 considered significant.

	Unicortical (n=95)	Bicortical (n=42)	Total	χ² (df)	p- value
Union					
Yes	86 (90.5%)	39 (92.9%)	125 (91.2%)	0.2 (1)	0.656
No	9 (9.5%)	3 (7.1%)	12 (8.8%)		

## Discussion

The fixation of medial malleolus fractures remains a pivotal aspect of ankle fracture management. While both unicortical and bicortical screw techniques are well-established [4], the choice between them hinges on factors such as mechanical stability, complication profile, and patient-specific characteristics.

In our study, no statistically significant difference in delayed union rates was observed between the unicortical (9%) and bicortical (7%) groups. This aligns with the findings of a recent retrospective cohort study published in the *Journal of the American Podiatric Medical Association* (JAPMA), which examined 126 patients and found no significant difference in complications, time to union, or return to full weight-bearing between the two techniques [5]. Both studies support the notion that, clinically, either fixation strategy can achieve satisfactory outcomes in appropriately selected patients.

A study by Pollard et al. (2010) [3] compared the pullout strength between 3.5-mm fully threaded bicortical screws and 4.0-mm partially threaded cancellous screws in medial malleolar fractures. The results indicated that bicortical screws provided significantly greater pullout strength, thereby offering more robust fixation.

Ricci et al. (2007) [6] conducted a biomechanical study using human cadaver specimens to evaluate the insertion torque generated during screw placement. Their findings revealed that bicortical lag screws generated an average maximum torque of 14.4 in-lbf, over three times greater than the 3.9 in-lbf generated by unicortical screws. This increased torque correlates with enhanced fracture site compression, contributing to improved stability.

Zabawa et al. (2025) [7] conducted a sawbone model study comparing single screw and two screw fixation in a bicortical fixation method. They concluded that in osteoporotic bone, two bicortical screw fixations are superior to single bicortical screws.

Richard et al. (2018) [8] compared single-screw fixation with two-screw fixation. The study did not show any difference and raises further questions about do we even need robust fixation for medial malleolus fracture.

The above-mentioned advantages are theoretically beneficial in scenarios involving poor bone quality or high biomechanical demands. However, despite these mechanical benefits, our data and that of the JAPMA study [5] suggest they may not result in measurable differences in short-term union or complication rates in typical fracture presentations. These convergent findings suggest that biomechanical superiority of bicortical screws may not necessarily translate into improved clinical outcomes. The absence of significant differences in union or complications suggests that surgeons can tailor fixation choice to patient-specific factors. Bicortical fixation may still be useful in patients with poor bone quality [9,10], comminuted fractures, or in revision cases where additional stability is desired. Conversely, unicortical fixation may be favored when minimizing operative time, or according to surgeon skill set and training.

Limitations

This study has several limitations. As a retrospective analysis, it is subject to selection bias and surgical decision-making may have been influenced by intraoperative findings not captured in the dataset. Additionally, functional outcomes and patient-reported satisfaction were not assessed, which limits the ability to correlate radiological healing with long-term ankle performance. The follow-up period, although sufficient to evaluate union, may not capture later complications such as hardware irritation or post-traumatic arthritis.

## Conclusions

This retrospective analysis demonstrates that both unicortical and bicortical screw fixation techniques are effective for the treatment of medial malleolus fractures, with no significant difference in union rates or early complication profiles. While biomechanical evidence favors bicortical fixation for its superior pullout strength and torque, these advantages did not translate into statistically improved clinical outcomes in our cohort. These findings suggest that fixation choice can be tailored to individual patient and fracture characteristics and surgeon skills. Further prospective studies are warranted to assess long-term functional outcomes and optimal indications for each method.
